# Sneezing and asymptomatic virus transmission

**DOI:** 10.1063/5.0019090

**Published:** 2020-07-01

**Authors:** Giacomo Busco, Se Ro Yang, Joseph Seo, Yassin A. Hassan

**Affiliations:** 1 Department of Nuclear Engineering, Texas A&M University, 3133 TAMU, College Station, Texas 77843, USA; 2 J. Mike Walker ’66 Department of Mechanical Engineering, Texas A&M University, College Station, Texas 77843, USA

## Abstract

The novel coronavirus disease (COVID-19) spread pattern continues to show that geographical barriers alone cannot contain a virus. Asymptomatic carriers play a critical role in the nature of this virus quickly escalating into a global pandemic. Asymptomatic carriers may transmit the virus unintentionally through sporadic sneezing. A novel Computational Fluid Dynamics (CFD) approach has been proposed with a realistic modeling of a human sneeze achieved by the combination of state-of-the-art experimental and numerical methods. This modeling approach may be suitable for future engineering analyses aimed at reshaping public spaces and common areas, with the main objective to accurately predict the spread of aerosol and droplets that may contain pathogens. This study shows that the biomechanics of a human sneeze, including complex muscle contractions and relaxations, can be accurately modeled by the angular head motion and the dynamic pressure response during sneezing. These have been considered as the human factors and were implemented in the CFD simulation by imposing a momentum source term to the coupled Eulerian–Lagrangian momentum equations. The momentum source was modeled by the measured dynamic pressure response in conjunction with the angular head motion. This approach eliminated the need to create an *ad hoc* set of inlet boundary conditions. With this proposed technique, it is easier to add multiple fixed and/or moving sources of sneezes in complex computational domains. Additionally, extensive sensitivity analyses based on different environmental conditions were performed, and their impact was described in terms of potential virus spread.

## I. INTRODUCTION

In light of ongoing events, the scientific community has been putting a great deal of effort in response to the 2019–2020 SARS-CoV-2 pandemic. Our role as a body of the engineering research community is to develop tools and engineer solutions that will be able to avoid or limit the future occurrence of the pandemic situations. As reported by the World Health Organization (WHO),^1^ the spread of the SARS-CoV-2 could be mainly due to respiratory droplets or airborne transmission (aerosol) and close contacts.^2^ It is extremely important to create a modern, reliable computational framework that is able to simulate different scenarios while containing as much physics as possible. This can be accomplished by the creation of a model that can quantify the number of droplets and aerosols evaporated and/or deposited on surfaces during all human related exhalations.

The pioneering work of Wells^3^ has been the first attempt to model and quantify the infective range of droplets expelled from the mouth or nose during coughing or sneezing. Wells addressed the main key factors that could be modeled and that could affect the spread of airborne infections. He developed the well-known droplet falling curve that was used to estimate the evaporation and deposition time of a single falling droplet. Xie *et al.*^4^ revisited the Wells droplet falling curve by considering the effect of relative humidity (RH), air speed, and respiratory jets. In their model, the droplets were able to diffuse in two dimensions. More complex models have been developed in recent years, for example, Redrow *et al.*^5^ introduced, in their coughing model, the physical properties of NaCl, amino acids, and lipids. Wei and Li^6^ studied closely the turbulence-related effects on droplet dispersion. Li *et al.*^7^ demonstrated the importance of considering heterogeneity on the humidity field when modeling the evaporation and dispersion of cough droplets. Other than the effect of RH, Conticini *et al.*^8^ claimed that atmospheric pollution may contribute to the high lethality of SARS-CoV-2 in Northern Italy. Chen and Zhao^9^ presented a detailed analysis on droplet dispersion in ventilation rooms.

On average, a healthy person sneezes four times^10^ per day and coughs^11^ two times per day. Coughing and sneezing have been addressed as the principal means of virus spreading mechanisms.^12,13^ Dbouk and Drikakis^14,15^ showed that the saliva droplets from coughing traveled a distance less than 2 m in the case of zero-wind conditions and the use of a face mask does not provide complete protection. Dbouk and Drikakis^14^ also suggested that the 2 m social distance may be insufficient considering the environmental conditions. Bhardwaj and Agrawal^16^ analyzed the chances of the survival of the virus present in the droplets based on the lifetime of the droplets under several conditions. They found that the chances of the survival of the virus are strongly affected by ambient temperature and humidity. They also explored the relationship between the drying time of a droplet and the growth rate of the spread of COVID-19 in five different cities and find that they are weakly correlated. The other means of asymptomatic spread have been proposed recently. Wang *et al.*^17^ showed how a massive upward transport of virus particles is observed in the case of toilet flushing, with 40%–60% of particles reaching above the toilet seat.

In this study, we focused our analysis on sneezing because it is by far the most violent spasmodic expiration of a mixture of moist air and saliva. Moreover, it has a potential of spreading infectious pathogens from asymptomatic carriers. Given the fact that sneezing happens to healthy people more frequently than coughing (*episodes/day*) in everyday life,^10,11^ asymptomatic carriers may transmit the virus unintentionally through sporadic sneezing. Furthermore, an increased number of sneeze episodes during the allergy season may also increase the risk of the asymptomatic spreading more likely.

We propose a novel Computational Fluid Dynamics (CFD) modeling approach that might suit future engineering analyses, which aims to create or reshape public spaces and common areas, with the main objective of accurately predicting the spread of aerosol and droplets that may contain the SARS-CoV-2 pathogen or any source of future airborne diseases. This study focuses mainly on the implementation of realistic initial and time-varying momentum source conditions for the human sneeze. In particular, we consider the angular head motion and time-intensity variation of the sneeze, a combination of effects that influenced the simulation results. The aforementioned set of initial conditions and time-varying momentum source came directly from an extensive set of experimental analyses performed at our thermal-hydraulics research laboratory.

To the best of knowledge, consideration of angular head motion during a human sneeze for CFD simulation has never been reported. The key features of the sneezing action were subdivided into two segments based on the experimental observations, the angular head motion and the dynamic pressure response during the sneeze transient, which were analyzed by image processing of high-speed videos and signal processing of the acquired data. Based on the analyzed data, the transient responses of the angular head motion and the dynamic pressure of the sneeze were then transformed into a mathematical model. These key features were introduced in the CFD model by adding a momentum source term to the coupled Eulerian–Lagrangian momentum equations. This approach eliminated the need to create an *ad hoc* set of inlet boundary conditions. With this proposed method, it will be easier to add multiple fixed or moving sources of sneezes in a complex computational domain such as multiple people sneezing in a crowded public area.

A comparison between our realistic approach and the widely used conventional CFD approach, characterized by no head motion, was performed. It has been shown that major differences are present in the predictions of both approaches, especially for the spread of the smallest Lagrangian particles with a diameter less than 10 *μ*m. The CFD approach proposed in this paper gave a more realistic estimate in terms of the spreading and evaporation of droplets and aerosols ejected during a sneeze. It was found that combining angular head motion and dynamic pressure response greatly increased the droplet cloud spread.

With the new model, we performed an extensive set of sensitivity analyses based on various environmental initial conditions. The role of relative humidity and ambient temperature has been addressed and compared in terms of cloud formation and spread. The role of different concentrations of suspended PM10 and PM2.5 particles in the atmosphere has been taken into consideration. This analysis guides in finding the best environmental conditions that could reduce the spread of the virus.

## II. EXPERIMENTAL METHOD

**FIG. 1.**
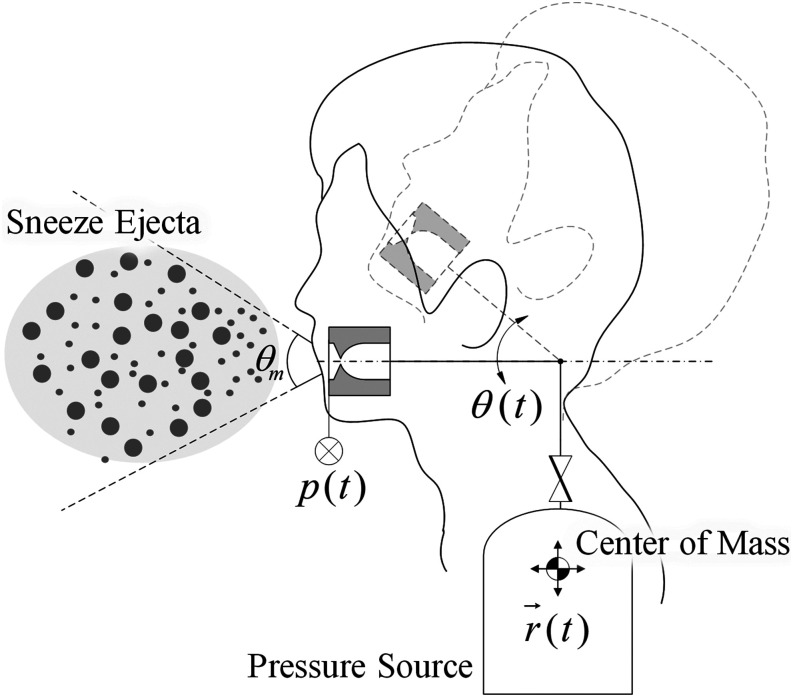
Human sneezing and spraying nozzle analogy.

Sneezing is a reflex mechanism of the respiratory system to prevent any undesired stimulus getting into the upper-respiratory tract. The main driving force of the human sneeze is the pressure induced by the spasmodic contraction of internal intercostal and abdominal muscles. A large pressure fluctuation during a short interval creates fast flow throughout the upper-respiratory tract, which breaks and entrains the mucus and saliva from the mouth cavity and eventually spraying them out to the atmosphere. From the engineering point of view, this sequential response of human sneezing draws an analogy to the spraying nozzle with a pressure source. Figure 1 illustrates the schematics of one to the human sneezing and spraying nozzle analogy.

In Fig. 1, the movement of the nozzle connected to a pressure source is overlaid on the illustration of a human sneezing. Consequently, to accurately model human sneezing, characterizing the pressure response *p*(*t*), nozzle movement as a consequence of head and body motion *θ*(*t*) and *r*(*t*), respectively, nozzle shape (shape of lips or mouth), and the mouth opening angle *θ*_*m*_ of ejecta treated as a mixture of aerosol and droplets during the transient is essential.

From the observation of multiple preliminary experiments of human sneezing, we found that the pressure response and the nozzle movement are the key time-varying parameters, while the nozzle shape and the spreading angle remained almost constant during the transient, and this was consistent with the findings of Gupta *et al.*,^18^ even though their investigation was for the case of a cough. Because these characteristics of human sneezing can be considered as the peer factors, generating a standard model of human sneezing as the initial and boundary conditions for accurate CFD simulation of experimental results using state-of-the-art measurement techniques should be the baseline for the realistic CFD simulation before we apply it to analyze further complex scenarios. By peer factors, we mean that the transient responses of human sneezing can vary per individual.

### A. Dynamic pressure response of a sneeze

**FIG. 2.**
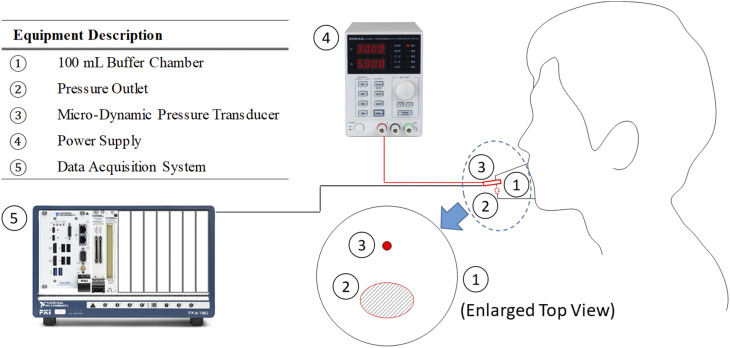
Pressure measurement.

As mentioned earlier, the driving force of the human sneeze is pressure. Thus, the dynamic pressure of human sneezing was measured using the state-of-the-art micro-dynamic pressure transducer (Kulite XCEL-100-50A) with 334.738 kPa full scale range and a maximum accuracy of ±0.5% output. Figure 2 depicts the experimental configuration for the dynamic pressure measurement.

**FIG. 3.**
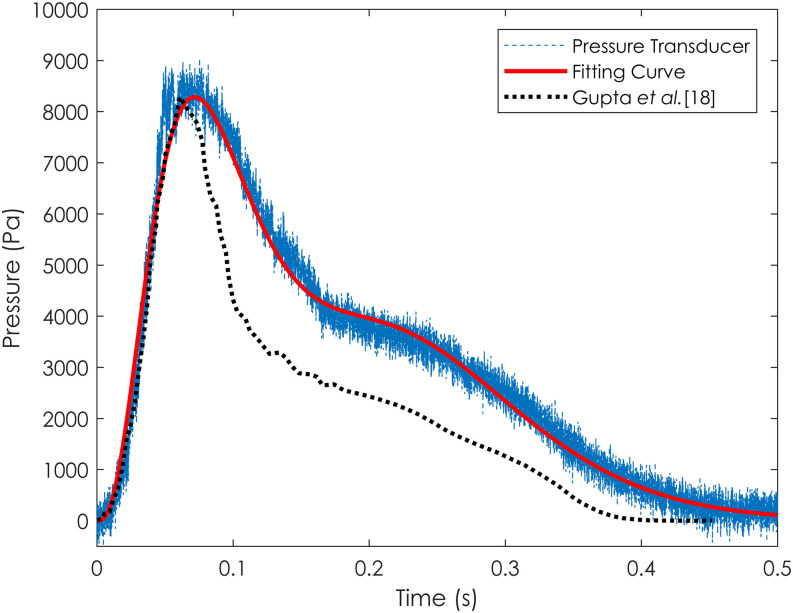
Dynamic pressure response of a human sneezing during the transient (standard deviation: 0.34 kPa).

The experimental configuration resembles that of Gupta *et al.*,^18^ while they measured the volumetric flow rate using a spirometer based on a Fleish type pneumotachograph. As shown in Fig. 2, a 100 ml buffer chamber was placed on top of the mouth cavity as the housing of the pressure transducer. An ellipsoidal pressure outlet with an area of about 120 mm^2^, which represents the mouth opening, was located on the buffer chamber. A Korad KA3005D adjustable DC power supply was employed as the power source for the pressure transducer. The pressure data were recorded by the full-bridge input channel with a sampling rate of 50 kHz on a National Instruments PXIe-6363 X Series DAQ connected to a PXIe-1092 chassis with a PXIe-8861 2.8 GHz quad core on-board controller. Figure 3 presents the experimental results of the dynamic pressure response of a human sneezing during the transient.

The measured data of Gupta *et al.*^18^ were the flow rate of a cough but not a sneeze. The flow rate data from Gupta *et al.*^18^ processed to a velocity data and then squared and normalized to match the dimension and peak value of pressure. Nevertheless, the sneeze pressure response envelope resembled that of the cough, as it can be modeled by the gamma-probability-distribution function as reported in the work of Gupta *et al.*^18^ The fitting curve of the present study is marked in red in Fig. 3, and it can be formulated using

$$p\left(t\right)=\left\{\frac{c_{1}t^{a_{1}-1}e^{-\frac{t}{b_{1}}}}{b_{1}^{a_{1}}\Gamma \left({\text{a}}_{1}\right)}+\frac{c_{2}t^{a_{2}-1}e^{-\frac{t}{b_{2}}}}{b_{2}^{a_{2}}\Gamma \left({\text{a}}_{2}\right)}\right\}\left(\text{Pa}\right),$$ (1)

where Γ is the gamma function, *a*_1_ = 4, *b*_1_ = 0.0235 s, *c*_1_ = 860.1073 Pa s, *a*_2_ = 9, *b*_2_ = 0.028 s, and *c*_2_ = 674.3917 Pa s. The R^2^ value of the fitting curve was 0.9937 with the standard deviation of 0.34 kPa.

### B. Flow visualization of human sneezing using high-speed cameras

**FIG. 4.**
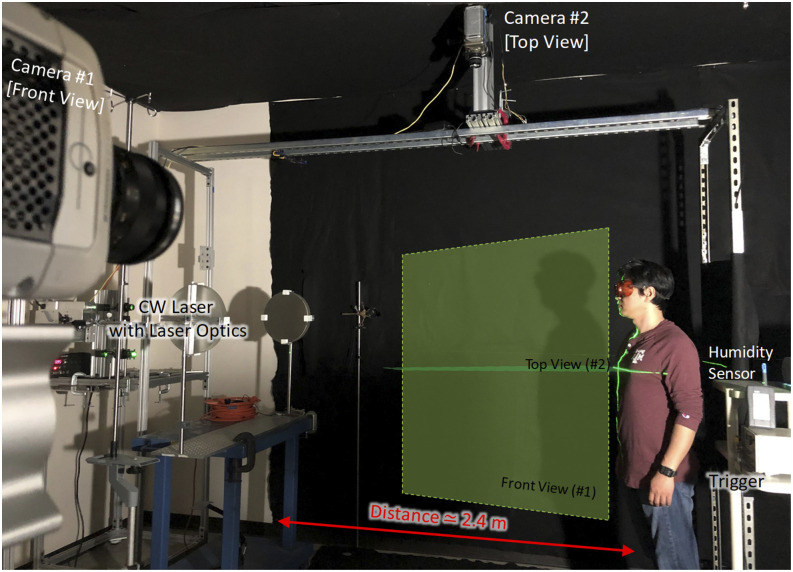
Experimental configuration for visualization of human sneezing.

In this section, an experimental method for flow visualization of human sneezing captured using high-speed cameras is presented. Figure 4 depicts the experimental configuration for the flow visualization.

As shown in Fig. 4, two high-speed cameras were mounted to capture the flow characteristics of the sneeze from the front view (camera No. 1, Phantom v711 with Zeiss Planar T^*^ 50 mm f/1.4 ZF.2 lens) and the top view (camera No. 2, Phantom v7.2 with Nikon NIKKOR 24 mm f/2.8 AI lens). A 20 W, 532 nm continuous-wave laser with a 50/50 beam splitter and two TSI Model 610026 light sheet optics generated the planar laser sheets for the front view (No. 1) and the top view (No. 2). The laser sheet for the front view was aligned vertically to the center plane of a human target, while the laser sheet for the top view was aligned horizontally at a height of 1.5 m. The frame rates of the high-speed cameras were set to 2000 fps. Room temperature and the relative humidity were measured by the portable humidity and temperature sensor Lufft C200.

**FIG. 5.**
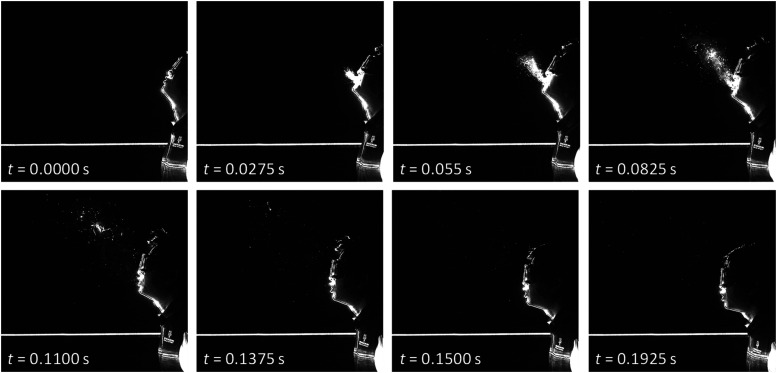
Snapshots of the sneezing action. Total duration: 0.1925 s.

To follow the CDC guidelines, the experimental setup was configured to allow one-man action with digital trigger using a Quantum Composers 9150+ pulse generator to initiate the high-speed camera recordings. The sneeze was induced by stimulation of the nasal mucous membrane of a healthy adult male. During the sneezing experiments, only one person who sneezes was allowed to be in the room, and all the surfaces were cleaned thoroughly with disinfectant after each sneeze. Figure 5 illustrates the sequential snapshots extracted from the high-speed images of the front view of the sneezing action (from camera No. 1 in Fig. 4). The total duration of the images was 0.1925 s with a time step of 0.0275 s.

The exhalation phase of the sneeze can be distinguished as two parts. The first part of the sneezing ejection contains a heterogeneous mixture of moist air (aerosols) and saliva droplets, which is presented in Fig. 5. These steps are characterized by a short time frame [0, *t*_*d*_] and can last about 0.2 s, where *t*_*d*_ stands for a droplet phase. As shown in Fig. 3, the pressure response peaked within this time frame, and most of the liquid phase ejection is expelled during this phase. This phase is followed by a second phase [*t*_*d*_, *t*_*a*_] where mostly remaining air from the lungs is exhaled, where *t*_*a*_ stands for an air exhalation phase. The total sneezing action can last about 0.5 s.

In addition to the pressure transient described in Sec. II A, the angular head motion is also a key parameter to describe human sneezing. To evaluate the realistic angular motion of the head, image processing of the consecutive images of the front view of human sneezing captured by the high-speed camera (from camera No. 1 in Fig. 4) was analyzed.

The angular head movement can be characterized by a whip-like motion. The angle *θ* of the face-mouth normal with respect to the horizontal direction has a decreasing phase (head down motion) followed by a slower increasing phase (head up motion) to go back to the head rest position. With a curve-fit of the experimental data, we were able to obtain the function of the angle *θ*(*t*),

$$\theta \left(t\right)=\left\{\begin{array}{cc} 40.71\cdot \sin\left(4.707\cdot t+1.764\right)+15.5\cdot \sin\left(13.46\cdot t+2.186\right), & \;0\leq t\leq t_{d}\\ 13.6865\cdot \log\left(t\right)+17.9155, & \;t_{d}<t\leq t_{a}. \end{array}\right.$$ (2)

**FIG. 6.**
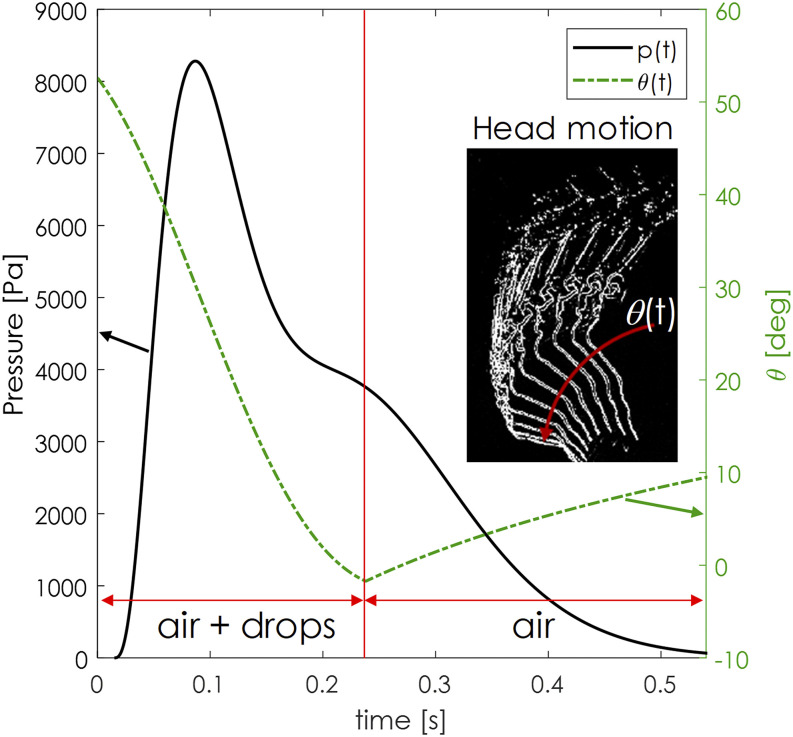
Pressure signal and angular time variation of the head. Separation in the exhalation phase is highlighted.

In this study, we used the values *t*_*d*_ = 0.2381 s and *t*_*a*_ = 0.5400 s that were determined from the pressure response and the image processing. Figure 6 shows the time evolution of the pressure signal and the function of the head angle movement.

These functions have been used to build the momentum source term for the CFD simulation. They have been treated independently and separately in the CFD implementation. Further details of the numerical methodology will be discussed in Sec. III.

### C. Measurement uncertainties

The measurement uncertainty of pressure is bounded by the dynamic pressure oscillation with the pressure transducer accuracy provided by the manufacturer, which is ±0.5% output. As shown in Fig. 3, the standard deviation of the pressure measurement was found to be 0.34 kPa, which is smaller than the pressure oscillation shown in the plot.

The angular movement of the head was extracted from the high-speed images, and the major source of uncertainty is the optical magnification factor *α*, which is a proportionality constant of the image magnification to physical space at the focal plane. The uncertainty quantification was performed in accordance with the *ITTC guide*.^19^ The high-speed camera was calibrated using the LaVision Type 204-15 3D Calibration Plate, and *α* = 1.056 mm/pixel for camera No. 1, which was used to measure the angular movement of the head. Other sources of uncertainty regarding the image detection can be the camera sensor (CMOS) distortion, lens distortion, and the normal view angle. In this study, the CMOS sensor distortion was neglected, and the image distortion by lens was assumed to be 4.97 pixels, which is 0.5% of the total length. The error of the normal view angle to the calibration plate was assumed to be 0.035 rad (2°). The calculated uncertainty for the magnification factor was found to be *σ*_*α*_ = 0.008 83 mm/pixel, which is ∼0.84% of the magnification factor. The angle can be calculated by the trigonometric identity with the known hypotenuse length *c* and opposite length *a* by *θ* = arcsin(*a*/*c*), and the propagation of uncertainty *σ*_*θ*_ can be expressed as follows:

$$\sigma_{\theta }=\frac{\sigma_{a/c}}{\sqrt{1-{\left(\frac{a}{c}\right)}^{2}}}=\frac{\left(\frac{a}{c}\right)\sqrt{{\left(\frac{\sigma_{a}}{a}\right)}^{2}+{\left(\frac{\sigma_{b}}{c}\right)}^{2}}}{\sqrt{1-{\left(\frac{a}{c}\right)}^{2}}}\approx 0.023^{○}/{\left.pixel\right|}_{\theta =60^{○},a=\frac{\sqrt{3}}{2},c=1}.$$ (3)

From image processing, the length measured for the angle calculation was 57 pixels. Thus, the maximum uncertainty of the angular head motion is ±1.33°.

## III. NUMERICAL METHOD

The physical problem is characterized by a large spectrum of space and time scales. A single droplet coming from the sneeze can have a diameter in the order of microns, while the largest scales of the outer environment can range up to tens of meters. The complete Eulerian description of the problem is very much impractical for this specific problem. Thus, a two-way Eulerian–Lagrangian approach is adopted for the present study. In this way, the dynamics of a single particle coming from the sneeze acts on a sub-grid scale and interacts only with the resolved Eulerian macro-scales by exchanging mass, momentum, and energy.

### A. Eulerian model

The continuous phase was modeled as a compressible homogeneous mixture between dry air and water vapor by solving a conservation equation for scalar variables that represents the mass fraction Y of each species in the mixture. This conservation equation is solved in addition to the global continuity equation. The material properties of the mixture are calculated as functions of mass fraction of the mixture species components. A given mixture property *ϕ*_mix_ has been calculated by mass-weighting the component property values,

$$\phi_{mix}=Y_{a}\phi_{a}+Y_{v}\phi_{v},$$ (4)

where *Y*_*a*,_ *Y*_*v*_ are the mass fractions and *ϕ*_*a*_, *ϕ*_*v*_ are the property values of air and water vapor, respectively. Since dry air and water vapor have been considered as a homogeneous mixture, we can assume that they share the same local velocity, pressure, and temperature. The two-way interaction of the continuous Eulerian phase and the dispersed Lagrangian phase has been achieved by taking into account the interphase mass, momentum, and energy exchange. The Reynolds number, based on the equivalent hydraulic diameter *D*_*h*_ = 4·*A/P*_*w*_ (*A* = mouth opening area and *P*_*w*_ = mouth opening perimeter) of the mouth opening and peak velocity, is approximately Re = 20 000. The turbulence Reynolds Averaged Navier–Stokes (RANS) realizable k-epsilon model has been adopted to close the turbulence problem. The relevant equations can be found in STAR-CCM+ v2019.2.1 manual.^20^ Relative humidity has been imposed as an initial condition by using the Antoine equation^21^ for the equilibrium pressure, and together with ambient temperature, it has been used for deriving the initial water and air mass fractions, respectively.

### B. Lagrangian model

The Lagrangian approach has been used to model the droplet spread of the sneeze. The particles were not directly resolved on the Eulerian field, but the interaction between the two phases was modeled. The mass, momentum, and energy of the Lagrangian phase could be exchanged with the continuous phase and vice versa. The analysis of the experimental data showed that the sneezing ejecta dynamics is characterized mainly by droplet evaporation and breakup. The observed physical phenomena from the experiments were implemented to our model.

The mass balance equation for the single particle is dictated mainly from droplet evaporation. The equation for mass balance of the particle *m*_*p*_ is in the following form:^22^

$$\frac{dm_{p}}{dt}=-\left(\frac{\rho_{p}D_{v}Sh}{D_{p}}\right)A_{s}\text{ln}\left(1+B\right),$$ (5)

where *ρ*_*p*_ is the density of the particle liquid phase, *D*_*p*_ is the molecular diffusivity of the liquid phase, *D*_*v*_ is the molecular diffusivity of the vapor phase, and *A*_*s*_ is the surface area of the particle, and we addressed the importance of a correct model for convective mass transfer by using a correlation for the Sherwood number (Sh) and the Spalding mass transfer number (*B*). The Ranz–Marshall^23^ correlation has been used for the Sherwood number closure model.

The momentum balance took into consideration several forces on the droplets. The mechanical forces taken into account were a drag force *F*_*D*_ and a buoyancy force *F*_*B*_,

$$m_{p}\frac{dv_{p}}{dt}=F_{D}+F_{B},$$ (6)

and the drag coefficient *C*_*d*_ for the drag force has been calculated from the Liu model.^24^

In addition, turbulent particle dispersion^25^ has been taken into account for the model by calculating the eddy turbulent time and length scales. A particle remains in the eddy until either the eddy time scale *τ*_*e*_ is exceeded or the separation between the particle and the eddy exceeds the length scale of the eddy *l*_*e*_. Both eddy time and length scale calculated from the RANS model are used to estimate the eddy velocity scale $u_{e}=\sqrt{2/3}l_{e}/\tau_{e}$. This velocity is used as the standard deviation for a normal (Gaussian) distribution with zero mean to randomly pick a particle velocity fluctuation to add to the instantaneous particle velocity *v*_*p*_. Once generated, a single realization of the velocity fluctuation continues to apply to a single particle until its eddy interaction time *τ*_*e*_ is exceeded.

The particle energy balance applied to the single particle took into consideration the convective heat transfer as follows:

$$m_{p}c_{p}\frac{dT_{p}}{dt}=FhA_{p}\left(T-T_{p}\right),$$ (7)

where *c*_*p*_ is the specific heat at constant pressure, *F* is the mass transfer correction that resolves the heat transfer reduction due to mass transfer,^26^ *A*_*p*_ is the surface area of the particle, *T*_*p*_ is the particle temperature, *T* is the surrounding fluid temperature, and *h* is the heat transfer coefficient calculated by the use of the Ranz–Marshall^23^ correlation.

The droplets’ distortion and breakup dynamics have also been taken into account by using the Taylor Analogy Breakup (TAB) model.^27^ The TAB model is based on Taylor’s analogy. The analogy represents a distorting droplet as a damped spring–mass system; it considers only the fundamental mode of oscillation of the droplet. The displacement and velocity of the mass in the spring–mass system correspond to the representative distortion and the rate of distortion quantities for the droplet. The TAB model accounts for the droplet shape oscillations, distortion, and breakup.

### C. Sneezing modeled as momentum source

From the analysis of several experimental sneeze runs, we were able to describe the dynamics of the human sneezing action. The key phenomena that characterize the sneeze are as follows:

(i) total duration of exhalation,
(ii) head movement,
(iii) pressure intensity variations, and
(iv) mouth shape and size,

which were used to build the sneezing momentum source term for the Eulerian phase. The instantaneous magnitude of the source term has been defined as

$$\left|S\left(t\right)\right|=p\left(t\right)/L,$$ (8)

where *p*(*t*) is the experimental pressure signal and *L* is the characteristic equivalent length of the human upper-respiratory system ducts. In the present model, we choose to leave pressure and the flow rate unconstrained. With reference to Eq. (8), we found that using a reference length L ≈ 0.3 m–0.6 m, equivalent to the length of the human chest/lung region, the induced exhaled air peak velocity was comparable with the experimental data. The horizontal and vertical components of the momentum source were calculated by considering of the time-varying angle *θ*(*t*),

$$\left\{\begin{array}{c} S_{hor}=\left|S\left(t\right)\right|\cos\left\{\theta \left(t\right)\right\},\\ S_{ver}=\left|S\left(t\right)\right|\sin\left\{\theta \left(t\right)\right\}. \end{array}\right.$$ (9)

**FIG. 7.**
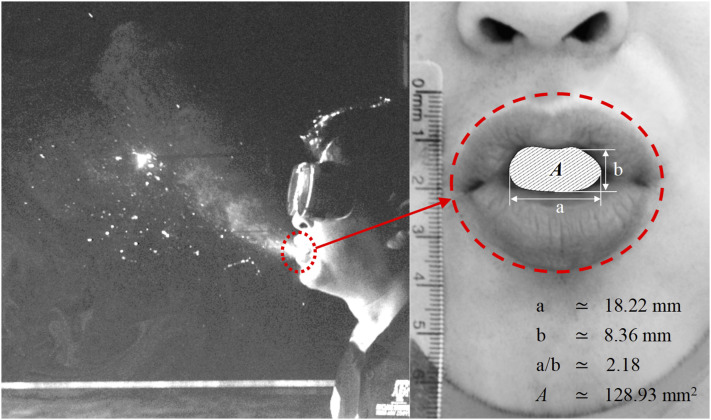
Mouth opening shape and dimensions.

The momentum source has only been applied within the mouth region of the computational domain. In this case, the region is a 3D rectangular region with the dimensions reported in Fig. 7, and the thickness of the volume region was assumed to be equal to the volume cell width of that particular region.

**FIG. 8.**
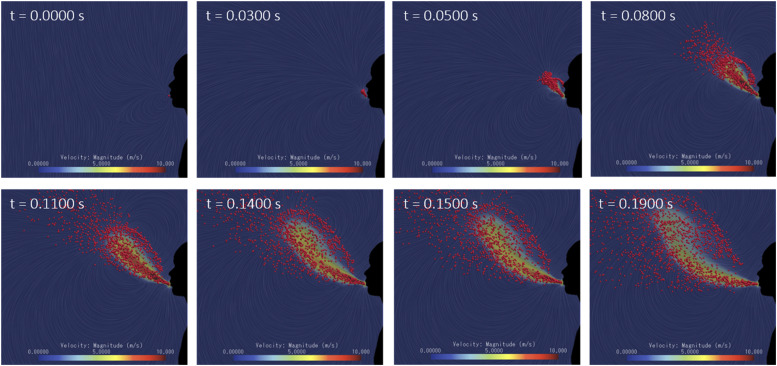
Snapshots of the modeled CFD sneezing action. Total duration: 0.19 s. Multimedia view: https://doi.org/10.1063/5.0019090.110.1063/5.0019090.1

It is worth stressing that the momentum source can be applied to any particular shape (e.g., circular and elliptical) that can resemble the mouth opening shape. The time frame of the application source term is from *t* = 0 to *t* = *t*_*a*_. The Lagrangian phase has been injected in the domain from a cone with a 60° opening, centered in the middle of the source term face, whose axis followed the time variation of *θ*(*t*). As a result, the complete movement generated by the sneezing action has been translated into our CFD model, as shown in Fig. 8 (Multimedia view).

**FIG. 9.**
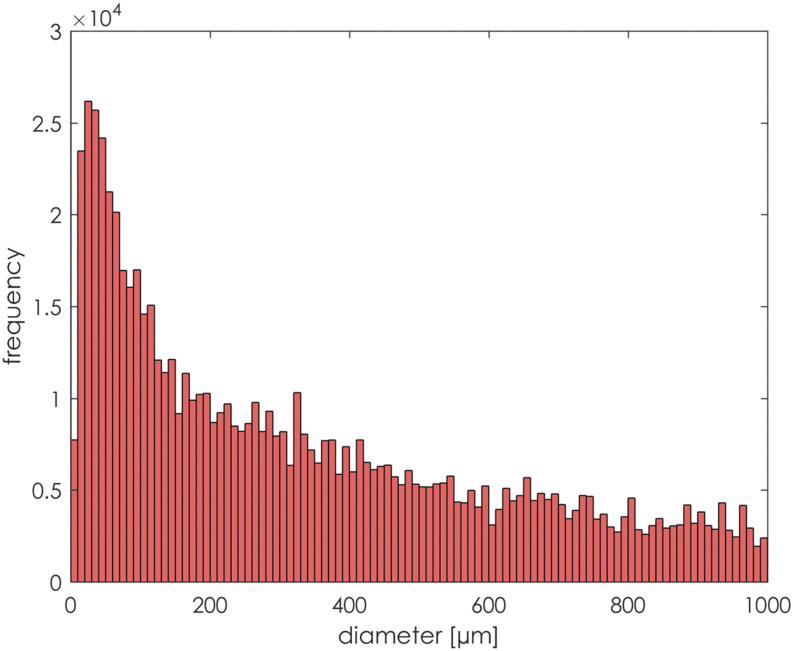
Imposed particle size distribution.

The particle size distribution is presented in Fig. 9. It followed the same log-normal distribution as proposed by Han *et al.*^28^ The time-varying injection velocity of the Lagrangian particles was equal to the maximum instantaneous air velocity *V*_max_(*t*) generated from the Eulerian momentum source.

### D. Computational domain and initial conditions

**FIG. 10.**
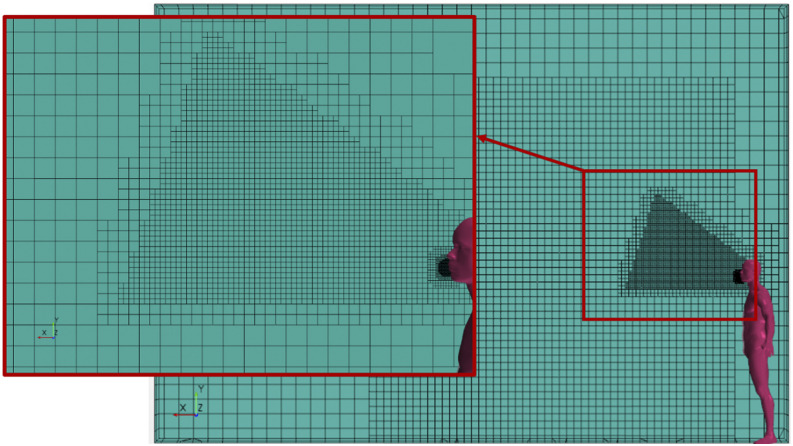
Computational mesh details.

The computational domain for the present analysis was a rectangular box with height *H* = 3.5 m, length *L* = 5 m, and width *W* = 2 m. As shown in Fig. 10, spatial discretization was performed with the automatic unstructured STAR-CCM+ trimmer mesher. Volume refinements were present in the momentum source region. The total number of volumes was N = 150 000.

Several initial conditions have been analyzed in the course of the study to investigate the effect of relative humidity on the sneeze cloud movement, evaporation, and deposition. Three ambient temperature cases were analyzed with *T* = 5 °C, *T* = 24 °C, and *T* = 35 °C, and for each temperature, three relative humidity (RH) values were considered as RH = 35%, RH = 65%, and RH = 95%. A total of 9 cases were analyzed. The simulated time duration of each run was *t*_*tot*_ = 50 s with a time step of Δ*t* = 5 × 10^−3^ s.

The influence on cloud dynamics for the presence of suspended PM10 and PM2.5 particles in the atmosphere has been studied. The concentration references for the PM10 and PM2.5 have followed the one from the United States Environmental Protection Agency (EPA). The EPA daily limit for PM10 particulate matter is 150 *μ*g/m^3^, and the limit for PM2.5 particulate matter is 35 *μ*g/m^3^. These limits were taken as reference for our simulation initial conditions. The Lagrangian phase of the PM particles could exchange only momentum with the coupled Eulerian phase. Two cases were studied based on the PM10 and PM2.5 particles’ concentrations. In the first case, we considered the nominal EPA’s concentration limits reported above. For the second case, we considered concentrations 10 times larger than the nominal EPA’s limits. The initial temperature for this analysis was set to *T* = 24 °C with a relative humidity of RH = 65%.

The maximum time window, for all the cases, has been chosen such that most of the particles in the simulations were either evaporated or deposited before the end of the simulation. The Lagrangian particles’ initial temperature was *T*_*p*_ = 35 °C in all the cases, and the total particle mass injected was equal to *m*_*p*_ = 5 mg.

### E. Comparison of the present model with traditional sneezing modeling approach

One additional case was simulated by using the most common CFD sneezing approaches found in the recent literature. This simulation was characterized by

(i) constant inlet velocity: *v* = 17 m/s and
(ii) constant sneezing angle: *θ*(*t*) = constant.

As can be found in previous studies, it was assumed that the head is fixed at a certain angle during sneezing and with a constant exhalation velocity. The inlet velocity was selected to equal the maximum peak velocity generated in our time-varying source term model. The angle between the sneezing axis and the horizontal direction was considered constant and equal to *θ* = −27.5°. The sneeze spreading angle was *α* = 25° as it can be found in the work of Dudalski.^29^ The values reported in his work have been averaged after an extensive literature review.

## IV. RESULTS

An exhaustive *a priori* Lagrangian droplet evaporation model validation has been performed before the setup of our CFD model. The analysis was followed by *a posteriori* comparison with our experimental data collected in terms of droplet spatial distribution, with initial conditions matching the laboratory environment. A complete sensitivity analysis followed based on several initial air temperature and humidity conditions. This analysis lead to the identification of the least favorable condition for sneeze cloud spread.

### A. Validation of the evaporation model

**FIG. 11.**
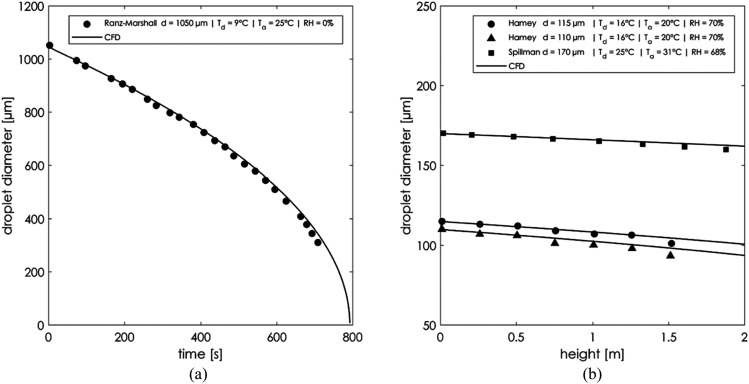
Droplet evaporation model validation: (a) Ranz–Marshall and (b) Hamey and Spillman.

The droplet evaporation model presented in Sec. III has been validated with some of the available experimental data. The validation of the CFD evaporation model was possible only for droplets of a diameter range bigger than 100 *μ*m. Figure 11 shows the validation results compared with two sets of experiments. The Ranz–Marshall^23^ experiment investigated the evaporation of motionless droplets in a dry environment with RH = 0%. The initial temperature of the droplet was *T*_*d*_ = 9 °C, the temperature of the surrounding air was *T*_*a*_ = 25 °C, and the initial droplet diameter was *d* = 1050 *μ*m. The validation results agreed closely with the diameter time evaporation curve. The data agreed well beyond our maximum CFD simulated time window of 50 s.

The results from the works of Hamey^30^ and Spillman^31^ from the *Cranfield Institute of Technology* have been compared in Fig. 11(b). Their work focused on the study of free-falling droplet evaporation in humid environments. The details of the experimental conditions are reported within the same figure. The CFD model reported good validation results in the droplet falling range of a human sneeze for a maximum of 2 m.

### B. Validation with present experimental data

**FIG. 12.**
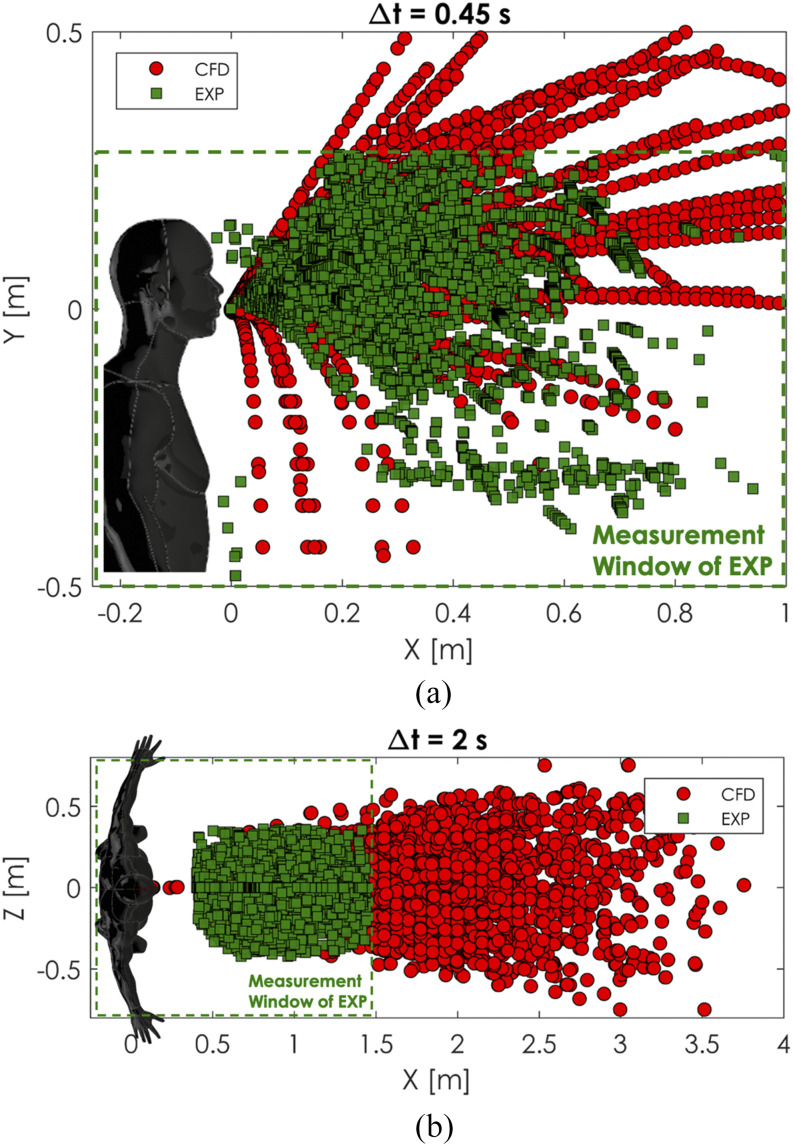
Comparison of cumulative time distribution of the sneezing droplets. Front view (a) and top view (b). Measurement window of the experiment (EXP) is the dashed line.

*A posteriori* validation analysis on the cumulative sneeze droplet distribution has been performed. The ambient initial conditions for the CFD simulation were the same as the experimental conditions. The laboratory temperature was at a constant temperature of *T*_*d*_ = 24 °C and a relative humidity of RH = 65%. The droplet distribution of several sneezes was tracked on the front view (camera No. 1 from the experiment) and the top view (camera No. 2 from the experiment), and the obtained results are shown in Fig. 12. The measurement window (marked in green dashed line in Fig. 12) of the experiment is smaller than that of the CFD simulation due to the limitation in the current optical setup. The measurement window for the front view was ∼−0.2 m–1 m in the x-axis and −0.5 m–0.3 m in the y-axis, and that of the top view was ∼−0.2 m–1.5 m in the x-axis and −0.7 m–0.7 m in the y-axis. To obtain the droplets’ trajectories, an image processing method similar to that of Bourouiba *et al.*^32^ was employed. The planar experimental domain had a smaller extension than the CFD domain. The cumulative time integration of the particle track on front view was Δ*t* = 0.45 s and Δ*t* = 2 s on plane 2. The CFD data have been extracted on the same planes inside a volumetric region of 1 mm thickness, which is the same as the experimental laser sheet.

Figure 12(a) compares the distribution recorded over a time window of 0.45 s, and Fig. 12(b) compares the distribution recorded over a time window of 2 s. Since the current resolution of the experimental images was 1.056 mm/pixel, we compared the CFD particles’ tracks of the droplets with a diameter larger than 1 mm only. The results show an exceptionally good comparison for both planes and time windows. This could mean that the entire mechanism of human sneezing involving the spasmodic contraction of internal intercostal and abdominal muscles can be efficiently modeled by providing an accurate pressure signal and head movement angle, which are the human factors entered in the CFD simulation.

**FIG. 13.**
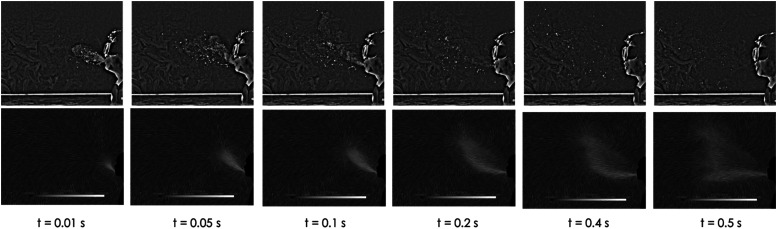
Cloud front propagation comparison. Multimedia view: https://doi.org/10.1063/5.0019090.210.1063/5.0019090.2

Figure 13 (Multimedia view) compares the sneeze front propagation between the CFD and the experimental images. The presence of theatrical fog highlighted the surrounding air induced motion rather than the ejected droplets phase. The combination of the head motion and air expiration generates a vertical cloud front of moist air that propagates in the horizontal direction. With the CFD analysis, we were able to observe the same induced air flow behavior and to understand better the physics of the problem. Several large scale vortices were generated and contributed to the flow mixing.

### C. Comparison of the new model and traditional model results

**FIG. 14.**
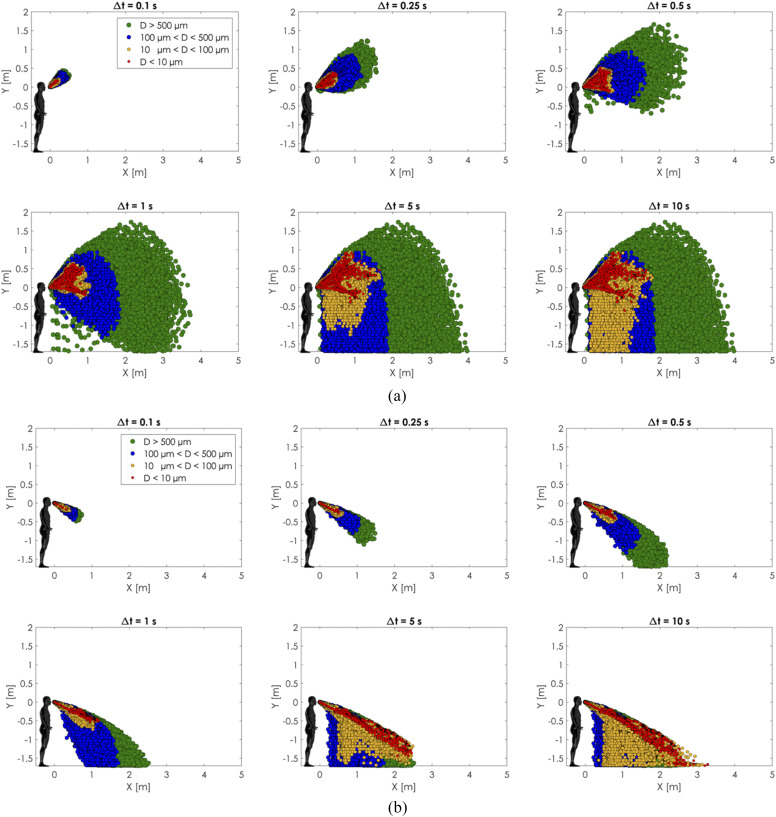
Comparison between (a) the present model with head motion and time-varying air expiration and (b) the conventional model with a fixed head angle and constant velocity. Front view comparison.

**FIG. 15.**
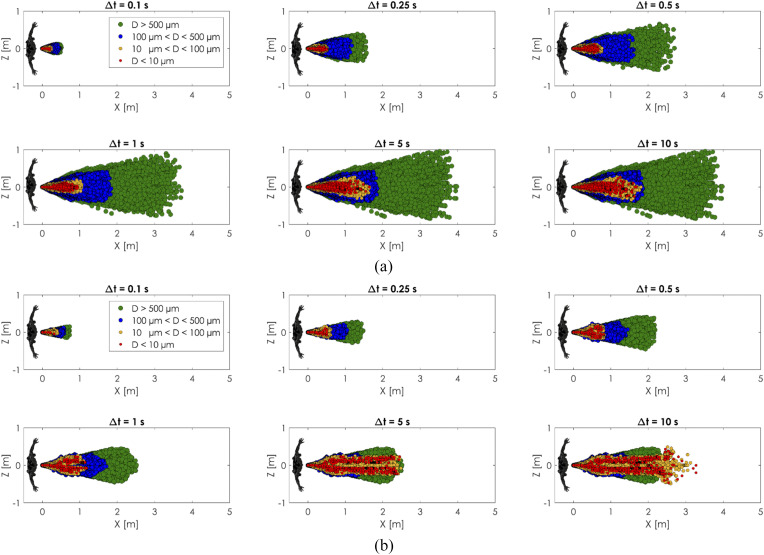
Comparison between (a) the present model with head motion and time-varying air expiration and (b) the conventional model with a fixed head angle and constant velocity. Top view comparison.

**FIG. 16.**
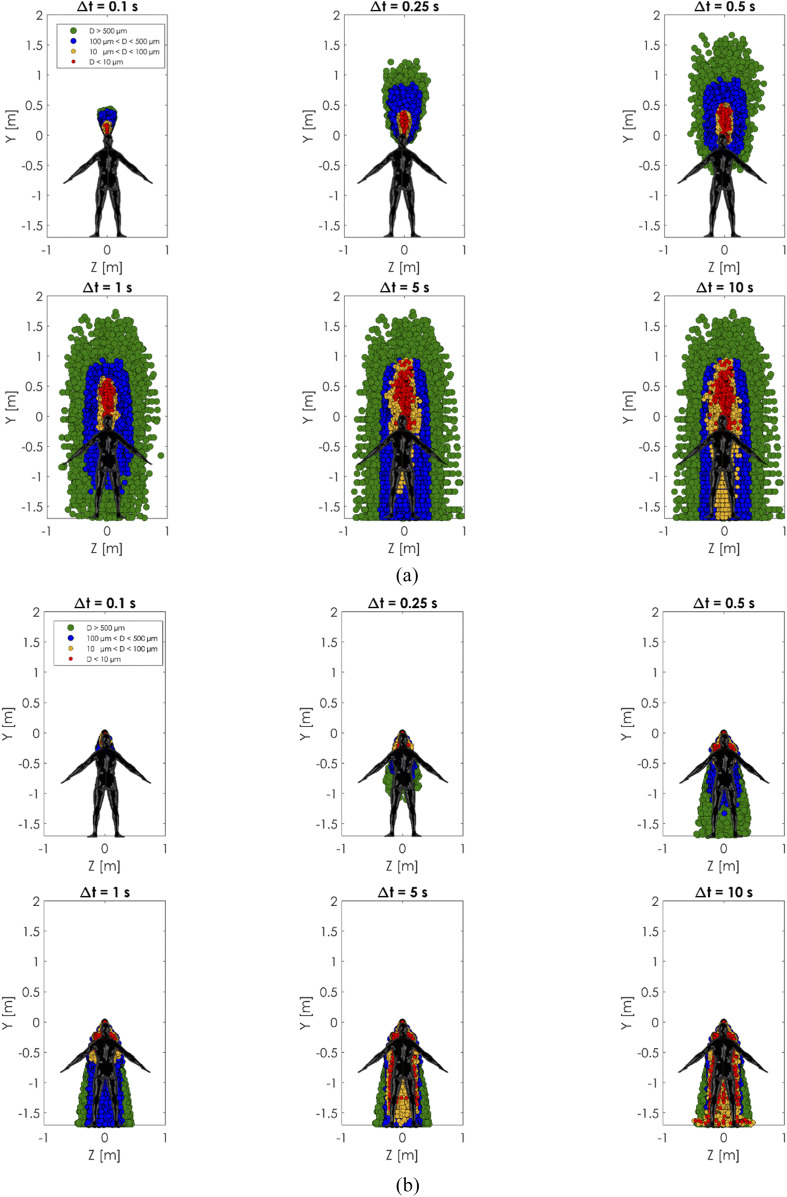
Comparison between (a) the present model with head motion and time-varying air expiration and (b) the conventional model with a fixed head angle and constant velocity. Right side view comparison.

The present realistic sneeze model and the conventional cough/sneeze model were comprehensively compared based on the spatial distribution and evaporation/deposition of the sneeze cloud, as shown in Figs. 14–16. The initial conditions of the simulations are the same as the experimental validation case reported in Sec. IV B.

Figures 14–16 show a comparison between the two approaches. Several time windows and cloud spread for different sizes of the droplets have been analyzed. The conventional approach with a *constant velocity/constant angle* showed less spreading of particles in all the cases. In the conventional cases, the particles covered a spatial region that was almost half of the present model cases. The cause of this discrepancy is mainly due to the moving angle and variable intensity condition imposed in the newly proposed model. The movement generated a whip-like motion that spread the particles more within the vertical direction.

The cloud dispersion analysis showed a maximum sneeze cloud range of 4 m in the downstream direction, 2 m in the lateral direction, and 2 m in the horizontal direction in the present study. The analysis showed how sneezing is by far the most violent spasmodic expiration of a mixture of moist air and saliva. The region of influence of the sneeze cloud is 2–4 times larger, in the case of zero-wind conditions, if compared to the coughing simulations of Dbouk and Drikakis.^14^ As mentioned in the Introduction, sneezing happens to healthy people more frequently than coughing (*episodes/day*) in everyday life,^10,11^ and asymptomatic carriers may transmit the virus unintentionally through sporadic sneezing. Furthermore, an increased number of sneeze episodes during the allergy season may also increase the risk of asymptomatic spreading more likely.

The results showed that even after 10 s from the onset of the sneeze, part of the PM10 airborne particles still did not deposit or evaporate. As we can observe in Fig. 13 (Multimedia view), the large air vortices generated from the combination of head motion and violent air expiration trap the floating PM10 particles at a higher elevation compared to the conventional model. The PM10 particles exchange their momentum with the large vortices, and their spatial mixing is enhanced. On the other hand, in the conventional model case, the fixed head angle and constant velocity let the PM10 particles to reach the ground faster with a limited spatial spread. Neither wind nor air recirculation was modeled in the simulations to focus on the sneeze-induced cloud motion. This means that in the presence of air recirculation or wind, the PM10 particles could potentially travel longer distances, which is also reported by Dbouk and Drikakis.^14^

After a person sneezes, an ejected saliva droplet could either evaporate completely before reaching a surface or simply deposit. The mass balance is reported in the following equation:

$$m_{total}=m_{deposited}+m_{evaporated}+m_{airborne}.$$ (10)

**FIG. 17.**
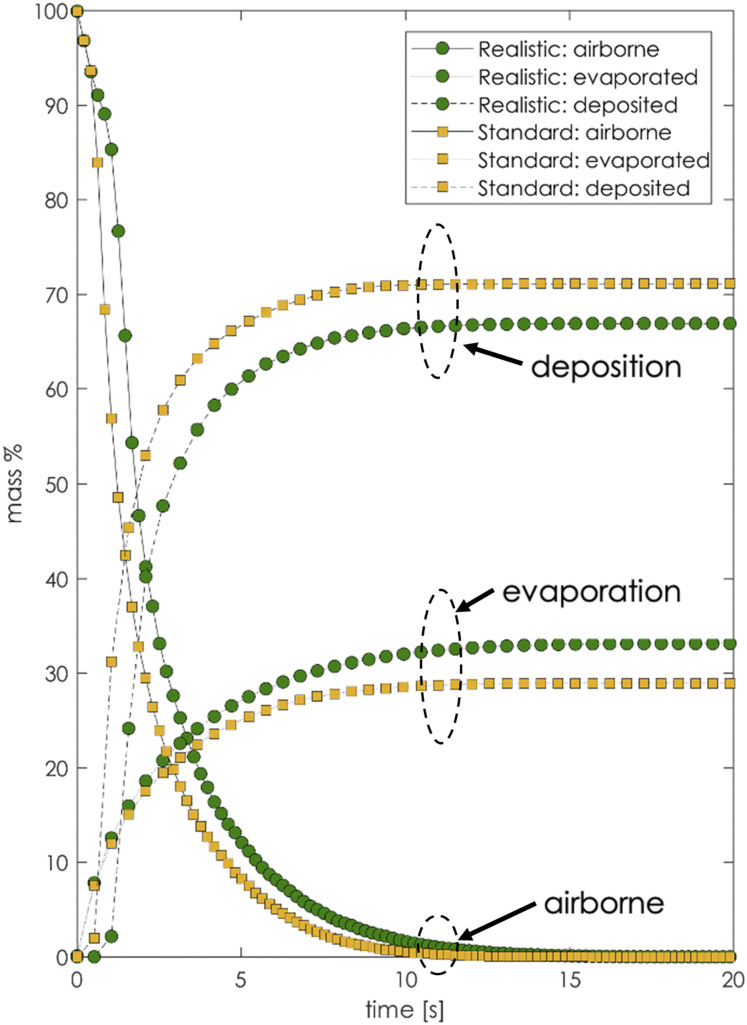
Evaporation, deposition, and residual airborne percentage of the initial ejected mass. Comparison of the conventional (standard) model with the present (realistic) model.

Figure 17 compares the percentage of the initial ejected total mass that has either evaporated, deposited, or remained in the air after a time window of 20 s. The comparison showed that the conventional model droplets either deposit or evaporate faster than the realistic case. In particular, most of the droplets in the conventional model deposited more on surfaces than the realistic case. At the same time, the droplets of the conventional case evaporated less than the realistic case.

### D. Humidity

**FIG. 18.**
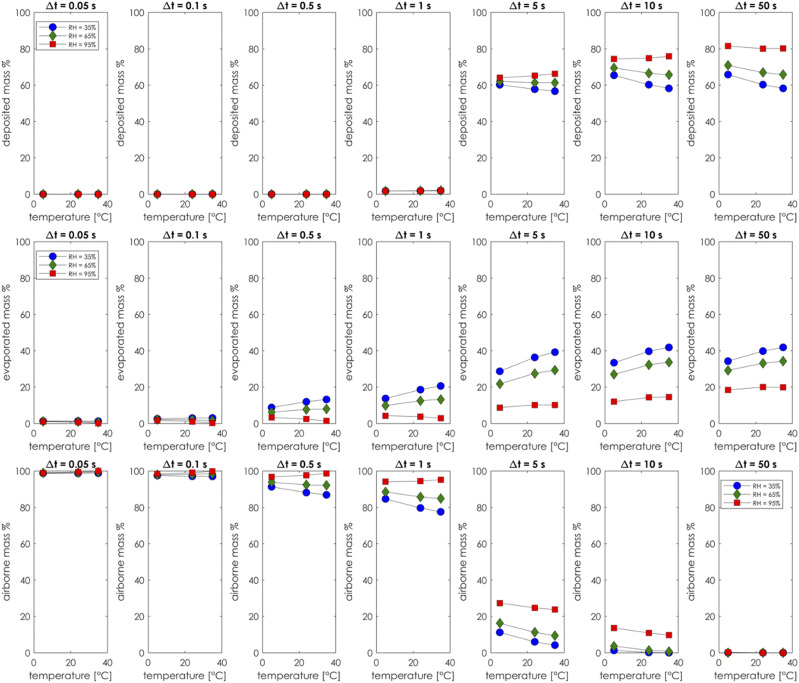
Deposition mass percentage for different time windows (top row). Evaporation mass percentage at different time windows (central row). Airborne mass residual percentage at different time windows (bottom row).

A sensitivity analysis based on the deposition/evaporation behavior has been performed under various relative humidity and environmental temperatures. Figure 18 summarizes the results of the analysis, and it shows the effect of both temperature and relative humidity on mass deposition and evaporation of the ejected particles. The airborne residual percentage has been defined as a particle mass that is still floating freely in the air and is neither evaporated nor deposited. The definitions of deposited, evaporated, and airborne masses are

$$deposited\;mass\;\%=\left(\frac{m_{deposited}}{m_{otal}}\right)\times 100,$$ (11)

$$evaporated\;mass\;\%=\left(\frac{m_{evaporated}}{m_{otal}}\right)\times 100,$$ (12)

$$airborne\;mass\;\%=\left(\frac{m_{airborne}}{m_{otal}}\right)\times 100.$$ (13)

The deposition, evaporation, and airborne residual curves are reported as a function of ambient temperature and relative humidity. Figure 18 shows a consistent trend between all the cases.

**FIG. 19.**
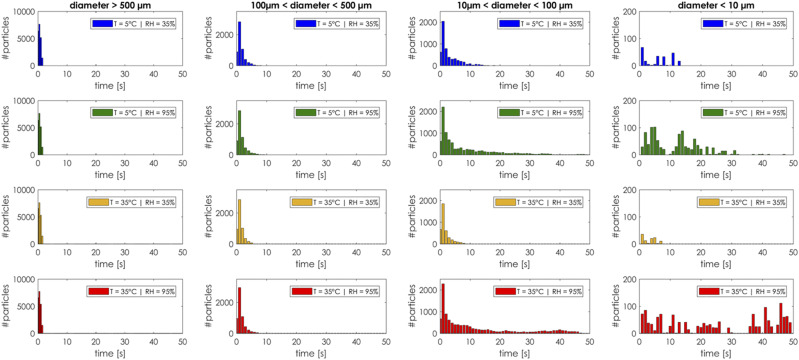
Number of droplets in air before evaporation or deposition as a function of droplet size, temperature, relative humidity, and time.

The central row of Fig. 18 reports the evaporation curve of all the cases. The results show that for all the time windows, the low humidity case consistently has the largest portion of mass evaporation. In contrast, the high humidity case always has the greatest percentage of particle deposition. The rapid decrease seen between the time windows Δ*t* = 1 s and Δ*t* = 5 s of the airborne residual percentage (bottom row) was due to the deposition (top row) of larger particles by gravity force, whereas the slower part of the decaying tail is due to the PM10 particles still floating in the air. Figure 19 shows the number of droplets in air before evaporation or deposition as a function of droplet size, temperature, relative humidity, and time. Once the droplet completely evaporates or deposits, it is removed from the particle count. We subdivided the droplets’ sizes in four groups: from very large (diameter > 500 *µ*m) to very small (diameter < 10 *µ*m). The largest droplets, regardless of the ambient conditions, fell on the ground within the same time interval (less than 10 s). This can be considered as a free falling of a spherical rigid body where the gravitational acceleration balanced with a drag force dominates the droplet trajectory. As the droplet diameter starts to decrease, buoyancy prevails, and the evaporation rate is the main driving mechanism for the cloud dynamics. In a cold and dry environment (*T* ≈ 5 °C, *RH* ≈ 35%), the evaporation rate is so fast that the number of the small volatile particles still in the air goes to zero within 15 s–20 s. In a humid and hot environment (*T* ≈ 35 °C, *RH* ≈ 95%), the evaporation rate is low and the number of the volatile particles still in the air does not go to zero after 50 s of simulation time.

Several conclusions can be drawn from this sensitivity analysis. For example, differences can be noted in sneezing in a cold, dry environment and sneezing in a very hot, humid environment. By looking at the plots above, we can state the following:

(i) In a cold and dry environment, there is a larger mass percentage of droplet evaporation. This, in turn, could leave all the nonvolatile airborne substances in the air that could spread indefinitely until deposited.
(ii) In a hot and humid environment, there is a larger deposition on surfaces and hypothetically less nonvolatile airborne substances left behind in the air. PM10 volatile droplets can still float in the air for a period longer than 50 s.

### E. Air particulate matter

**FIG. 20.**
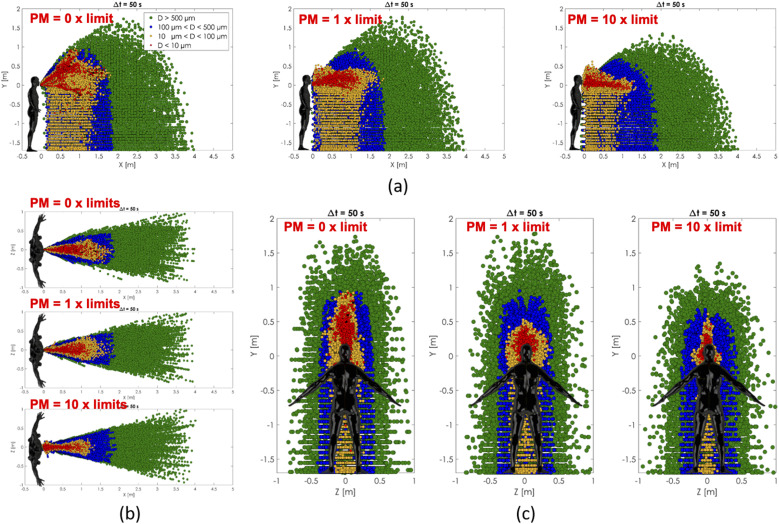
Cloud spread influence on PM10 and PM2.5 particles at different concentrations. Front view (a), top view (b), and right side view (c).

The effect of particulate matter in the atmosphere has been taken into account for our sensitivity analysis. Two different PM10 and PM2.5 concentrations have been considered simultaneously in our simulations. The EPA daily limit for PM10 particulate matter is 150 *μ*m/m^3^, and the limit for PM2.5 particulate matter is 35 *μ*m/m^3^. In the following analysis, the labels “*0x limit*” refer to zero suspended PM particles, “*1x limit*” refer to nominal EPA’s daily limits concentrations, and “*10x limit*” refers to values 10 times greater than the daily EPA’s limits. The effect on the cloud movement and dispersion can be observed in Fig. 20. The particle size distribution after 50 s in space has been reported as a function of PM concentration limits.

The overall effect on cloud dispersion for the presence of PM10 and PM2.5 particles was mainly related to the spatial extension of the cloud. Less volumetric dispersion and an increased concentration of the sneeze cloud were observed as the PM concentration increased.

**FIG. 21.**
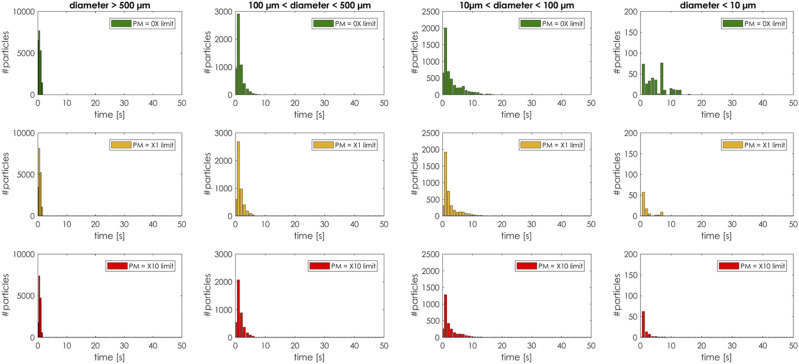
Number of droplets in air before evaporation or deposition as a function of droplet size, particulate matter concentrations, and time.

Figure 21 shows the number of particles of a given diameter still in the air as a function of particulate matter concentration. The effect of an increased PM concentration is to decrease the number of particles in the air more quickly.

**FIG. 22.**
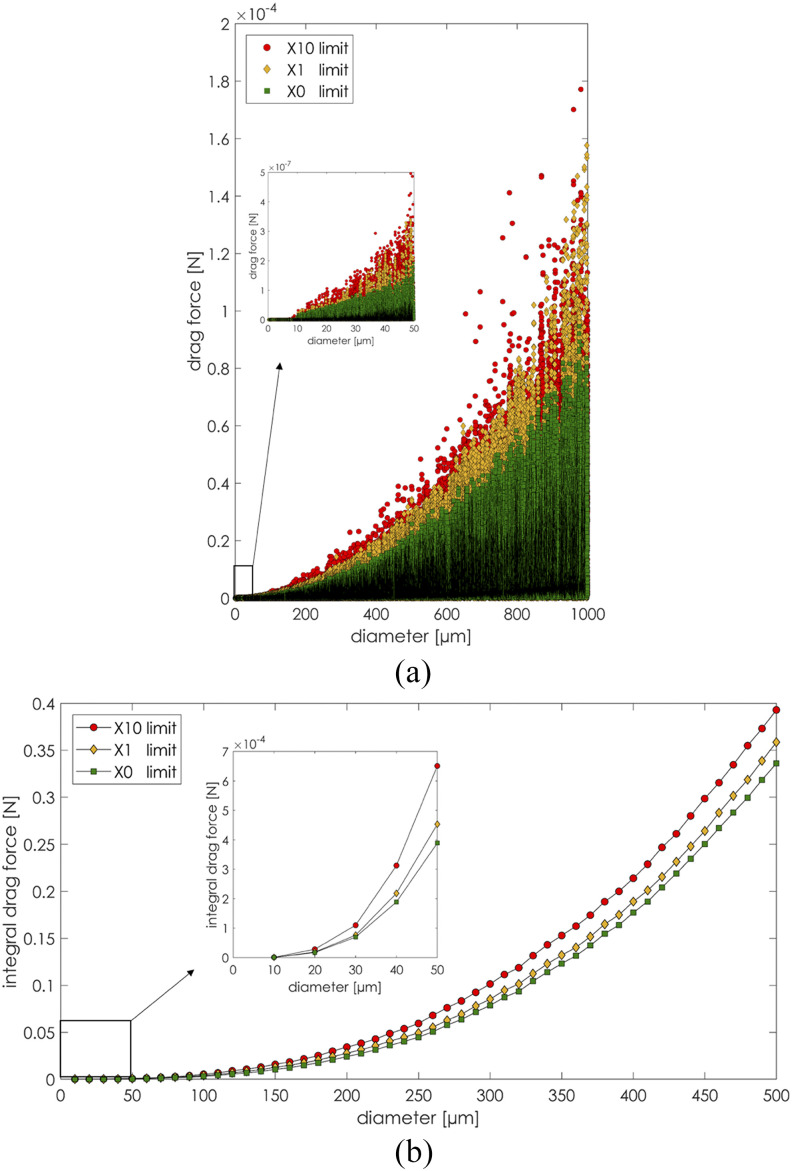
Particles’ drag force. Drag force spectrum (a) and integral drag force (b).

The increase in particulate matter concentration in the air increased the overall drag exerted on the sneezing particles across the entire diameter spectrum. In particular, the PM particles influenced the continuous phase, which, in turn, influenced the sneezing droplets. Figure 22(a) reports the magnitude of the drag acting throughout the entire simulation on every particle as functions of their diameters. For each fixed particle diameter, the upper limits of the drag force spectrum were always higher for the largest PM10 and PM2.5 particles’ concentration. Figure 22(b) shows the integral drag force magnitude acting on a given set of sneeze particles’ diameter *δ*. The integral drag force has been defined as

$$F_{iD}\left(\delta \right)=\int_{0}^{\delta }\left|F_{D}\left(\delta \right)\right|d\delta .$$ (14)

From Fig. 22, the overall increase in drag in the presence of higher PM concentrations can be deducted. The increased drag force for high PM concentrations reduced the particles’ spatial spread and increased the particles’ surface deposition rate. More specifically, for the high PM concentration, the large droplets deposited faster than the clean air condition. The spatial concentration of the small sneeze droplets increased because of higher drag forces.

## V. CONCLUSION

Extensive computational studies of the human sneeze have been conducted with realistic modeling achieved by the combination of state-of-the-art experimental and numerical methods. This deepened out the understanding of the dynamics of the sneeze and provided a set of realistic data that were provided by our CFD model.

The model validation aligned well with the experimental data, meaning that the entire mechanism of a human sneeze can be effectively modeled by providing transient responses of an accurate pressure signal and a head motion angle. In addition to the constant mouth opening angle and area, those time-varying variables can be considered as the human factors entering the CFD simulation.

The present approach has been compared with the conventional model currently found in the literature. Our proposed model showed that the conventional approach gives, in general, more confined results. The comparison of the two models showed that the conventional model cloud spread in space and time occupies a volume that is almost half of the present model. The cloud spread analysis of a sneeze showed a maximum zone of influence of 4 m in the downstream direction, 2 m in the lateral direction, and 2 m in the horizontal direction.

The analysis showed how sneezing is by far the most violent spasmodic expiration of a mixture of moist air and saliva. The region of influence of the sneeze cloud is 2–4 times larger, in the case of zero-wind conditions, if compared to the coughing simulations of Dbouk and Drikakis.^14^ Given the fact that sneezing happens to healthy people more frequently than coughing in everyday life,^10,11^ asymptomatic carriers may transmit the virus unintentionally through sporadic sneezing. Furthermore, an increased number of sneeze episodes during the allergy season may also increase the risk of the asymptomatic spreading more likely.

The conventional model droplets either deposited or evaporated faster than the realistic case. The droplets of the conventional model evaporated less and deposited more than the realistic case. The role of humidity and ambient temperature has also been considered, and the sensitivity analysis showed that in a cold and dry environment, there is a larger percentage of droplets’ evaporation. This, in turn, could leave all nonvolatile airborne substances in the air, spreading indefinitely until deposited on a surface. In a hot and humid environment, there is an increased particle deposition on surfaces, and hypothetically, less nonvolatile airborne substances are left behind. The influence of air particulate matter PM10 and PM2.5 on sneeze cloud dispersion showed less volumetric diffusion and an increased spatial concentration of the sneeze cloud as the PM concentration increased. The main reason for the less volumetric diffusion of the sneeze cloud was the increase in the sneeze droplets’ drag as the PM concentration increased.

With the proposed method, it will be easy to add multiple fixed or moving sources of sneezes in a complex computational domain such as multiple people sneezing in a crowded public area. This approach would be beneficial to assess and analyze the pathogen spreading from the human sneezing action using CFD, especially for indoors with known heating, ventilation, and air conditioning configurations to design and reshape the interior structures.

## DATA AVAILABILITY

The data that support the findings of this study are available from the corresponding author upon reasonable request.
